# The quest to find TLC: Tendril-less cucumbers, that is

**DOI:** 10.1093/plcell/koae137

**Published:** 2024-05-02

**Authors:** Vicky Howe

**Affiliations:** Assistant Features Editor, The Plant Cell, American Society of Plant Biologists; Department of Developmental Genetics, Heinrich-Heine University, Düsseldorf 40225, Germany

If you have ever grown cucumbers (*Cucumis sativus*) at home, you will know just how unruly these plants can be. As the primary stem grows and produces leaves from each side of the shoot apical meristem, branches and tendrils emerge from axillary meristems located at the leaf axils, forming a sprawling vine that will take over your garden if left untamed. In a commercial context, these disorderly tendrils and branches block airflow and light and divert resources away from fruit production. They must therefore be painstakingly removed, increasing both labor costs and the risk of infection through the resulting wounds. An ideal solution would be to have cucumber plants that develop fruit at the leaf axils without producing branches or tendrils. However, breeding such plants via targeted approaches requires an understanding of the molecular mechanisms governing both tendril and branch formation.

It is fortunate then, that **Junjun Shen and coauthors** (Shen et al. 2024) have uncovered some key players in the regulation of tendril and branch formation, which could prove to be the genetic targets agricultural science has been looking for. In this study, Shen and colleagues screened a library of retrotransposon insertion mutants and found one lacking tendrils. The transposon disrupts a gene encoding a GRAS transcription factor that the group designated *CsTL* (for tendril-less), resulting in a complete lack of tendril initiation (see Figure).

**Figure.**
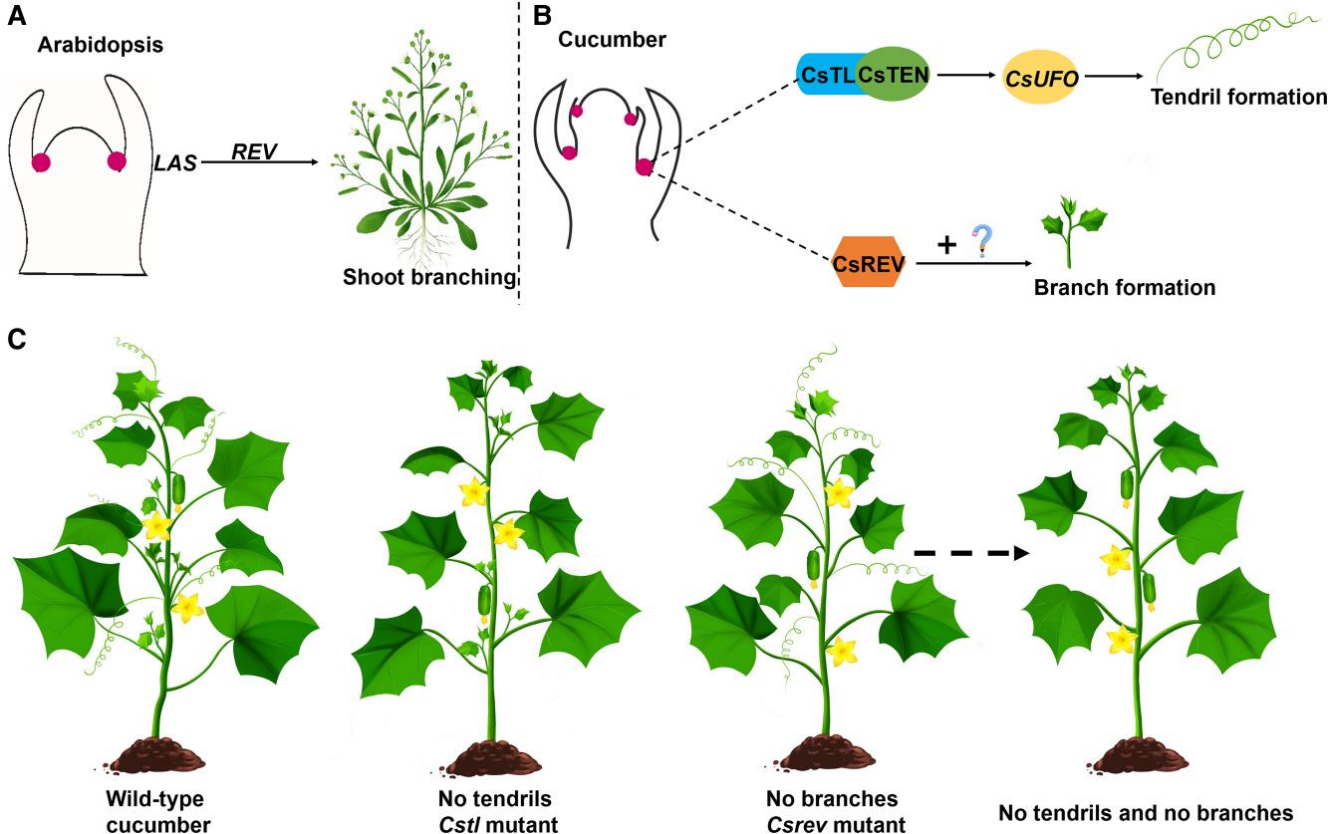
Altering cucumber tendril and branch formation. Unlike Arabidopsis, where **(A)** the TL homolog LAS functions upstream of *REV* to control shoot branching, **(B)** cucumber TL interacts with CsTEN to activate transcription of *CsUFO*, which regulates tendril formation. A second pathway involving *CsREV* is involved in branch formation in cucumber. **C)** Mutating both *CsTL* and *CsREV* might produce cucumber plants lacking tendrils and branches. Reprinted from Shen et al. (2024), Figure 7.

To identify the downstream targets of CsTL, the authors looked for genes differentially expressed between wild-type and *CsTL* knockout plants. Two transcription factors with decreased transcriptional expression in the *CsTL* knockout plants, *TENDRIL* (*CsTEN*) and *UNUSUAL FLORAL ORGANS* (*CsUFO*), stood out among the candidates: *CsTEN* was mutated in another tendril-less cucumber line (Wang et al. 2015), and *CsUFO* mutants produced abnormal tendrils (Chen et al. 2021; Hong et al. 2023). The group reasoned that CsTL might transcriptionally activate *CsTEN* and *CsUFO* by binding to their promoters. Instead, they found that CsTL physically interacted with CsTEN, which in turn enhanced CsTEN-mediated expression of *CsUFO* to promote tendril development (see Figure).

Interestingly, while the CsTL homologs in Arabidopsis, rice (*Oryza sativa*), and tomato (*Solanum lycopersicum*) all have conserved functions in branching, knocking out or overexpressing *CsTL* in cucumber perturbed tendril development without affecting branching. In Arabidopsis, the CsTL homolog LATERAL SUPPRESSOR (LAS) functions upstream of *REVOLUTA* (*REV*) to promote shoot branching (see Figure). However, knocking out *CsREV* in cucumber resulted in plants with defective branching but normal tendril initiation. This suggests that while *CsREV* has a conserved function in controlling branching across species, *CsTL* function has diverged from the other model plants.

A comparison of the Arabidopsis and cucumber genomes revealed that although both the *TL* and *REV* genes were duplicated in a whole-genome duplication event occurring in the Cucurbitaceae family (Guo et al. 2020), the duplicated copies were likely lost in cucumber. *CsREV* retained its function in branch regulation while *CsTL* gained a new function in regulating tendril formation. This is in contrast to a fellow Cucurbitaceae member, watermelon (*Citrullus lanatus*), where the *TL* homolog, *CILs*, controls the development of not just tendrils but also branches and flowers (Jiang et al. 2023).

Thus, this study reveals that, unlike previously studied species, cucumber has 2 distinct pathways regulating branching and tendril formation. It provides 2 potential gene targets to eliminate branching and tendrils in cucumber: *CsTL* and *CsREV*. Simultaneous deletion of the 2 genes might improve cucumber architecture, with the potential to increase yields and reduce labor input. Perhaps this is just what I need in my garden.

## Dive Curated Terms

The following phenotypic, genotypic, and functional terms are of significance to the work described in this paper:
